# Interprofessionalism as a driver for professional nursing education aimed at comprehensive care for sexual and gender minorities by 2030

**DOI:** 10.1590/0034-7167-2024-0195

**Published:** 2025-09-01

**Authors:** Gabriela Rodrigues Bragagnollo, Bruno Pereira da Silva, Tauani Zampieri Fermino, Débora de Souza Santos

**Affiliations:** I Universidade de Campinas. Campinas, São Paulo, Brazil; II Universidade de São Paulo. Ribeirão Preto, São Paulo, Brazil

**Keywords:** Interprofessional Education, Integral Healthcare Practice, Sexual and Gender Minorities, Sustainable Development, Public Health Nursing, Educación Interprofesional, Práctica Integral de Atención, Minorías Sexuales y de Género, Desarrollo Sostenible, Enfermería en Salud Pública

## Abstract

**Objectives:**

to reflect on the contribution of interprofessional education in nursing to the comprehensive care of sexual and gender minorities, aligned with the Sustainable Development Goals (SDGs) of the 2030 Agenda.

**Methods:**

a theoretical-reflective study grounded in the principles of interprofessionalism, focusing on SDGs 4, 5, and 10.

**Reflections and Discussions:**

interprofessional education in nursing is essential for promoting inclusive and equitable care that respects cultural and identity diversities, while ensuring the inclusion of sexual and gender minorities in health services. These approaches are crucial to reducing structural inequalities, improving access to care, and strengthening social justice.

**Conclusions:**

interprofessional and interdisciplinary education in nursing is essential to transcend fragmented models of health education, fostering comprehensive and inclusive care that addresses the needs of sexual and gender minorities.

## RESUMEN

**Objetivos:**

reflexionar sobre la contribución de la formación interprofesional en enfermería para el cuidado integral de las minorías sexuales y de género, en alineación con los Objetivos de Desarrollo Sostenible de la Agenda 2030.

**Métodos:**

estudio teórico-reflexivo fundamentado en los principios de la interprofesionalidad, con énfasis en los Objetivos de Desarrollo Sostenible 4, 5 y 10.

**Reflexiones y Discusión:**

la formación interprofesional en enfermería es esencial para promover un cuidado inclusivo y equitativo, respetando las diversidades culturales e identitarias, además de garantizar la inclusión de las minorías sexuales y de género en los servicios de salud. Estas estrategias son cruciales para reducir las desigualdades estructurales, mejorar el acceso a la atención y fortalecer la justicia social.

**Conclusiones:**

la formación interprofesional e interdisciplinaria en enfermería es fundamental para superar los modelos fragmentados de educación en salud, promoviendo un cuidado integral e inclusivo que responda a las necesidades de las minorías sexuales y de género.

## INTRODUCTION

We are part of a vast territory with significant socioeconomic and cultural regional differences that shape and influence the health-disease process, creating a complex and challenging healthcare landscape in Brazil. To address these inequalities, the Brazilian Federal Constitution of 1988 established and institutionalized social rights, including universal, equitable, and comprehensive access to healthcare services. This marked a pivotal shift toward justice and full citizenship in what had been an exclusive and unequal healthcare system.

In the pursuit of a more inclusive healthcare system, valuing cultural diversity in care settings becomes fundamental. In 2001, the United Nations Educational, Scientific and Cultural Organization (UNESCO) reaffirmed in the Universal Declaration on Cultural Diversity that cultural diversity is part of humanity’s heritage and inseparable from respect for human dignity. According to UNESCO, cultural diversity is essential for preserving cultural identities, especially in a globalized world where cultural homogenization is often imposed^(1)^.

The Universal Declaration of Human Rights (UDHR), adopted by the United Nations General Assembly on December 10, 1948, is a cornerstone document in the field of human rights. Comprising a preamble and 30 articles, it outlines the fundamental rights and freedoms inherently entitled to all human beings, regardless of race, color, religion, sex, language, political or other opinion, national or social origin, property, birth, or any other status.

While the UDHR does not specifically address contemporary issues such as gender identity and sexual orientation, its universal principles support the fight for inclusive and comprehensive care. In alignment with these values, the World Health Organization’s (WHO) “Agenda 2030”, launched in 2015, includes Sustainable Development Goals (SDGs) focused on promoting human rights, gender equality, and cultural diversity^(2)^.

Understanding culture as a factor that can either promote inclusion or perpetuate exclusion is essential. Cultural norms and traditions, often influenced by religious values, can reinforce oppression and hinder health equity, particularly for sexual and gender minorities^(3)^.

In this context, Primary Health Care (PHC) plays a crucial role in reducing health inequities by providing coordinated and continuous care tailored to the specific needs of these populations. In Brazil, PHC is guided by principles such as longitudinality, comprehensiveness, and community orientation, which, when effectively implemented, enable comprehensive biopsychosocial care^(4)^.

PHC also stands out in the realm of sexual and reproductive health, offering actions ranging from the prevention and treatment of sexually transmitted infections to supporting transgender individuals with hormonal and surgical interventions. To ensure comprehensive care and equitable services, interdisciplinary and interprofessional approaches are indispensable. Collaboration among healthcare professionals is fundamental to delivering inclusive care that addresses the specific needs of these populations^(4)^.

Within Brazil’s Unified Health System (SUS in Portuguese), promoting health equity requires policies that address the needs of vulnerable populations, ensuring fair and effective access to services^(5)^. Nurses, as essential members of PHC teams, must be adequately trained to serve sexual and gender minorities, ensuring comprehensive care that respects and addresses their realities.

Recognizing diversity as a key factor in promoting health in its broadest sense underscores the importance of equity within the SUS. In Brazil, equity is a foundational principle for fostering social justice, going beyond formal equality of access to guarantee that everyone’s needs are met according to their specific circumstances. This involves addressing the unique needs of populations in vulnerable situations, ensuring that health policies and actions contribute to reducing inequalities and advancing social justice^(6)^. In this sense, PHC, by providing continuous and coordinated care, plays a pivotal role in diminishing health inequities^(7)^.

The Health Equity Promotion Policies within the SUS comprise governmental programs and actions aimed at respecting diversity and delivering comprehensive care. Their goal is to eliminate unjust disparities among population groups by considering variables such as social class, ethnicity, gender, and sexual orientation^(6)^. Brazil has sought to promote cultural diversity and sexual and gender rights through policies, programs, and guidelines, such as the National Plan for Women’s Policies, Inclusive Education Programs, anti-discrimination legislation, support for LGBTQIA+ events, training programs for health and education professionals, strengthening LGBTQIA+ Comprehensive Health Policies, and promoting inclusive workplaces.

These initiatives reflect the State’s commitment to diversity and inclusion. However, their effectiveness remains a challenge, as the implementation and enforcement of these policies vary across the country. Progress requires continued engagement from the government, civil society, and individuals to foster a culture of respect and equality.

Thus, understanding the social representations that permeate daily life and care practices is critical to promoting equity. Interprofessional collaboration is an essential strategy to ensure that the training and practice of healthcare professionals align with the principle of equity and the goals of Agenda 2030.

## REFLECTIONS AND DISCUSSIONS

Comprehensive healthcare for the LGBTQIA+ population extends beyond technical and scientific knowledge. It requires a critical understanding of the social, cultural, and political dimensions that influence both access to and the quality of care.

In Brazil, the National Policy for Comprehensive LGBT Health (PNSI-LGBT), established in 2011, aims to reduce inequalities and promote respect and inclusiveness^(7)^. However, its implementation continues to face significant challenges due to the persistent impact of social stigmas and hetero-cisnormative practices within healthcare services.

The inequalities faced by the LGBTQIA+ population are rooted in cultural and historical processes that perpetuate structural disparities. For nursing education to contribute effectively to promoting equity, curricula must critically address the sociocultural and historical influences on gender and sexuality. Social norms and cultural representations not only shape perceptions of health and disease but also influence the training and practices of healthcare professionals^(8)^.

A nursing education grounded in interprofessional principles is essential for overcoming fragmentation in health education^(9)^. This approach fosters a deeper understanding of the challenges faced by sexual and gender minorities, equipping professionals to provide inclusive and equitable care.

Studies indicate that many training programs still incorporate hetero-cisnormative perspectives, limiting healthcare professionals’ ability to deliver truly equitable care. To address these shortcomings, nursing curricula must challenge cultural and social biases regarding gender and sexuality. They should emphasize the impact of social norms on the health of minorities and prepare future professionals to respond to these needs with sensitivity and inclusivity^(10-12)^.

### Comprehensive care and LGBTQIA+ Diversity: challenges and strategies

Comprehensive healthcare for the LGBTQIA+ population requires a broad approach that extends beyond technical knowledge. It incorporates a critical understanding of the social, cultural, and political dimensions that influence the health-disease process, as well as access to and the quality of care. Such care must align with the principle of comprehensiveness, which transcends a disease-focused approach to address the complexity of each individual’s needs, respecting their specificities and contexts.

To enhance understanding and accessibility, it is crucial to disseminate broader concepts of gender and sexual diversity, including the term “cisgender” (or “cis”). This terminology helps professionals recognize the impact of hetero-cisnormative frameworks on clinical practice and fosters an inclusive environment for non-hegemonic identities. Practices such as using gender-neutral language and respecting individuals’ chosen names are essential for ensuring care that upholds the individuality and dignity of LGBTQIA+ individuals^(8,13)^. However, these practices are often overlooked, underscoring the need for training that challenges limiting social norms to enable truly comprehensive care.

National and international studies, including those conducted in Brazil and Australia, reveal that LGBTQIA+ individuals face significant barriers to accessing healthcare services, such as discrimination and alienation by professionals-consequences of prejudice and disrespectful attitudes^(14,15)^.

These challenges are rooted in a history of structural inequalities and exclusionary social practices. Healthcare professionals must develop a nuanced understanding of how cultural and social norms shape perceptions of health and care practices. For example, a study in Santa Catarina, Brazil, examining the experiences of transgender individuals and nursing teams across different levels of care, found that inadequate training in sexual and gender diversity contributes to care practices that perpetuate exclusion^(11)^.

Academic curricula, often influenced by hetero-cisnormative perspectives, restrict the understanding of diverse ways of being and living, thereby limiting the ability to provide genuinely comprehensive and equitable care. Addressing health inequities requires integrating critical insights into the cultural, religious, and historical influences that affect the health of sexual and gender minorities into the education of nurses and other healthcare professionals. Incorporating content on the historical trajectory of oppression and its impact on health is a powerful educational strategy for promoting equity and humanization in healthcare practices^(13,16)^.

This critical focus is vital because studies show that traditional training, steeped in hetero-cisnormative norms, continues to fall short in adequately preparing professionals to deliver inclusive and equitable care to minorities. Furthermore, training programs that encompass sexual and gender diversity and provide practical experiences with historically marginalized groups are essential for achieving comprehensive care.

Integrating intergroup contact practices, particularly with sexual and gender minorities, is a strategy for fostering empathy and understanding of the unique realities shaping these groups’ experiences. An intersectional perspective, which considers overlapping oppressions such as racism, sexism, and LGBTQIA+ discrimination, is also indispensable for healthcare professionals to develop a holistic approach to care. This perspective enables professionals to recognize and respect the multiple identity dimensions and life contexts of individuals.

Comprehensive care requires healthcare professionals to be prepared to understand and address each individual’s needs in a contextualized and critical manner. This necessitates training that fosters the development of welcoming and care practices grounded in empathy, respect, and inclusion. Such training must aim to overcome the limitations imposed by traditional social norms, ultimately promoting truly inclusive and equitable healthcare services.

### Interprofessional education: inclusive perspectives on care for the lgbtqia+ population

Interprofessionality involves the development of collaborative and integrated practices among healthcare professionals from diverse fields. Through interprofessional education, essential competencies for healthcare practice extend beyond technical skills to include listening, engaging in dialogue, and sharing responsibilities^(9)^.

This approach equips professionals to understand that comprehensive care, particularly for vulnerable populations such as the LGBTQIA+ community, requires the recognition and appreciation of diverse knowledge and experiences. It fosters interdependence and complementarity among professionals, preventing subordination and ensuring that no single discipline dominates care approaches.

Interdisciplinarity, meanwhile, highlights the importance of integrating knowledge that transcends the boundaries of individual disciplines. This approach is fundamental to addressing complex health issues situated within broader cultural, social, and economic contexts^(9)^. For example, the health of LGBTQIA+ populations necessitates a critical understanding of gender, sexuality, and equity. To promote equitable and inclusive healthcare practices, education must encourage the critical analysis of social and cultural norms that sustain prejudice and inequalities, often rooted in hetero-cisnormative paradigms^(11,13)^.

Interprofessionality and interdisciplinarity align with the National Curriculum Guidelines (DCNs) and the National Policy on Permanent Health Education (PNEPS). These policies encourage universities to create spaces for reflective and dialogical educational practices, addressing training gaps through dialogue between diverse areas of knowledge and practice.

Programs such as VER-SUS (2004), Pró-Saúde (2007), and PET-Saúde (2010) exemplify the role of interprofessionality in challenging stereotypes and prejudices, promoting education focused on equity. These programs foster interaction between diverse areas of knowledge and healthcare practices, preparing students and professionals to meet the needs of LGBTQIA+ populations with empathy, technical competence, and relational sensitivity. By integrating diverse teams, they enhance the capacity of future professionals to deliver more inclusive and comprehensive care^(13)^.

It is also essential to reflect on the role of culture in perpetuating exclusionary and segregating processes, often rooted in Christianity. These cultural norms have historically upheld forms of oppression that influence societal perceptions of sexuality and gender. Overcoming health inequities thus requires the deconstruction of these norms and the incorporation of a critical understanding of the social and cultural influences on minority health^(13)^.

To meet the SDGs outlined in the 2030 Agenda, healthcare education must integrate interprofessional dimensions starting at the undergraduate level. This focus is particularly relevant in PHC, where professionals often serve as the first point of contact for vulnerable and marginalized populations requiring continuous and comprehensive care.

Interprofessional educational practices should be emancipatory and oriented toward social justice, enabling future professionals to understand the impact of social and cultural conditions on health and respond inclusively. Universities should function as spaces for constructing transformative practices, with curricula designed to train professionals committed to inclusion and equity. This foundation is vital for building a healthcare system that serves everyone.

This approach directly supports the SDGs, which emphasize the importance of education tailored to global health needs. To provide care that considers the whole person, universities must promote environments of dialogue and interprofessional practice, fostering the development of critical education aimed at social justice. This model transforms professional practice and contributes to a more inclusive and equitable healthcare system, particularly addressing the needs of the LGBTQIA+ population.

Cultural diversity, a central aspect of health promotion, should be emphasized in Brazilian public policies to ensure equitable access to healthcare for all, with special attention to gender and sexual minorities. Nursing plays a crucial role in this context. Higher education in nursing has evolved to respond to historical circumstances, fostering therapeutic and interprofessional care relationships that require the critical and creative construction of knowledge. This process must include ongoing dialogue among individuals, families, and other members of the healthcare team.

It is equally important to acknowledge that individuals are part of a broader society, making it essential to integrate collective health into nursing education. Recognizing that social phenomena emerge through interactions and roles in the world underscores the need for universities to engage in discussions about their social function. Such reflections enable the development of emancipatory educational alternatives. Curricula that overlook social complexity and diversity compromise a comprehensive understanding of the health-disease process and hinder teamwork capabilities, reducing the effectiveness of care delivery^(13,16)^.

Therefore, curricular content must be approached critically and contextually, fostering education that addresses social demands and ensures the right to health for all, particularly the most vulnerable populations. This underscores the importance of interprofessionality and interdisciplinarity in nursing education, preparing professionals to work collaboratively and effectively. By adopting this approach, it becomes possible to promote global and equitable health in alignment with the SDGs of the 2030 Agenda.

## FINAL CONSIDERATIONS

Interprofessional and interdisciplinary education in nursing is essential to move beyond fragmented models of health education, fostering comprehensive and inclusive care that addresses the needs of sexual and gender minorities. By integrating knowledge and practices from various fields, this approach enhances professionals’ capacity to navigate the social, cultural, and ethical complexities involved in caring for the LGBTQIA+ population. Thus, future nursing professionals are better prepared to provide care that respects diversity and ensures health equity.

Incorporating these dimensions into nursing curricula is crucial to ensuring an education that is sensitive to the specific needs of this population. This alignment with the SDGs of the 2030 Agenda, particularly those related to quality education, gender equality, and reducing inequalities, strengthens professional practice and contributes to the creation of a more just and equitable healthcare system capable of delivering respectful and humanized care.

Therefore, it is imperative that nursing curricula include interprofessional educational practices addressing the health needs of this population. Training programs should encourage collaboration across different areas of knowledge and consider the multifaceted realities of sexual and gender minorities to achieve health equity. By doing so, comprehensive care can be effectively realized within the context of SUS, respecting individual specificities and promoting health in an inclusive manner.
