# Supplementary material for: Interprofessionalism as a driver for professional nursing education aimed at comprehensive care for sexual and gender minorities by 2030
Source: Rev Bras Enferm. 2025 Sep 1;78(Suppl 1):e20240195. doi: 10.1590/0034-7167-2024-0195 (PMC12407867; doi:10.1590/0034-7167-2024-0195)
Supplement: Translation

# Translation: A interprofissionalidade como indutora da formação profissional em enfermagem para o cuidado integral das minorias sexuais e de gênero até 2030

**DOI:** 10.1590/0034-7167-2024-0195pt

**Authors:** Gabriela Rodrigues Bragagnollo, Bruno Pereira da Silva, Tauani Zampieri Fermino, Débora de Souza Santos

**Affiliations:** I Universidade de Campinas. Campinas, São Paulo, Brasil; II Universidade de São Paulo. Ribeirão Preto, São Paulo, Brasil

**Keywords:** Educação Interprofissional, Prática Integral de Cuidados de Saúde, Minorias Sexuais e de Gênero, Desenvolvimento Sustentável, Enfermagem em Saúde Pública

## Abstract

**Objetivos:**

refletir sobre a contribuição da formação interprofissional na enfermagem para o cuidado integral às minorias sexuais e de gênero, alinhado aos Objetivos de Desenvolvimento Sustentável da Agenda 2030.

**Métodos:**

estudo teórico-reflexivo, embasado nos princípios da interprofissionalidade, com foco nos Objetivos de Desenvolvimento Sustentável 4, 5 e 1.

**Reflexões e Discussões:**

a formação interprofissional na enfermagem é fundamental para promover um cuidado inclusivo e equitativo, respeitando as diversidades culturais e identitárias, além de garantir a inclusão das minorias sexuais e de gênero nos serviços de saúde. Essas abordagens são cruciais para reduzir as desigualdades estruturais, melhorar o acesso ao cuidado e fortalecer a justiça social.

**Considerações Finais:**

a formação interprofissional e interdisciplinar em enfermagem é essencial para transcender os modelos fragmentados de educação em saúde, promovendo um cuidado integral e inclusivo que responda às demandas das minorias sexuais e de gênero.

## INTRODUÇÃO

Formamos um imenso território, com importantes diferenças regionais socioeconômicas e culturais, que singularizam e determinam o processo saúde-doença, cenário que compõe a complexidade e o desafio do cuidado à saúde na realidade brasileira. Visando diminuir as desigualdades, com a promulgação da Constituição Federal de 1988, o Estado brasileiro reconheceu e deu força institucional aos direitos sociais, como o acesso universal, igualitário e integral às ações e serviços de saúde, que passam a refletir o ideal de justiça e de cidadania plena em um sistema de saúde outrora excludente e desigual.

Nessa busca por um sistema de saúde mais inclusivo, a valorização da diversidade cultural nos espaços de cuidado torna-se fundamental. Assim, em 2001, a Organização das Nações Unidas para a Educação, a Ciência e a Cultura (UNESCO) reafirmaram, através da Declaração Universal sobre a Diversidade Cultural, que essa diversidade é um patrimônio da humanidade e parte inseparável do respeito à dignidade humana. Segundo a UNESCO, a diversidade cultural é um mecanismo essencial para a preservação das identidades culturais, especialmente em um mundo onde a globalização tende a impor uma homogeneização cultural^(1)^.

A Declaração Universal dos Direitos Humanos (DUDH) é um documento fundamental no campo dos direitos humanos, adotado pela Assembleia Geral das Nações Unidas em 10 de dezembro de 1948. Composta por um preâmbulo e 30 artigos, delineia os direitos e liberdades fundamentais a que todos os seres humanos são inerentemente intitulados, independentemente de raça, cor, religião, sexo, língua, opinião política ou de outra natureza, origem nacional ou social, propriedade, nascimento ou qualquer outro status.

Apesar de a DUDH não tratar especificamente das questões atuais, como identidade de gênero e orientação sexual, seus princípios universais sustentam a luta por um cuidado inclusivo e integral. Em consonância com esses valores, a “Agenda 2030” da Organização Mundial da Saúde (OMS), lançada em 2015, inclui metas de desenvolvimento sustentável focadas na promoção dos direitos humanos, na igualdade de gênero e na valorização da diversidade cultural^(2)^.

Entender a cultura como um elemento que pode tanto promover a inclusão quanto perpetuar a exclusão é essencial. Normas culturais e tradições, muitas vezes influenciadas por valores religiosos, podem reforçar opressões e prejudicar a equidade em saúde, especialmente para minorias sexuais e de gênero^(3)^.

Nesse cenário, a Atenção Primária à Saúde (APS) desempenha um papel crucial na redução das iniquidades em saúde, proporcionando cuidados coordenados e contínuos que atendem às necessidades específicas dessas populações. No Brasil, a APS se fundamenta em princípios como longitudinalidade, integralidade e orientação comunitária, que, quando devidamente fortalecidos, viabilizam um cuidado biopsicossocial abrangente^(4)^.

A APS também se destaca na saúde sexual e reprodutiva, oferecendo ações que vão desde a prevenção e o tratamento de infecções sexualmente transmissíveis até o apoio a pessoas transgênero, que necessitam de orientações sobre intervenções hormonais e cirúrgicas. Para garantir a integralidade do cuidado e promover um atendimento equitativo, abordagens interprofissionais e interdisciplinares são indispensáveis. A articulação entre diferentes profissionais de saúde é fundamental para a efetivação de um cuidado inclusivo e adaptado às necessidades específicas dessas populações^(4)^.

No contexto do Sistema Único de Saúde (SUS), a promoção da equidade em saúde exige políticas que atendam às necessidades das populações vulneráveis, assegurando um acesso justo e eficaz aos serviços^(5)^. Nesse sentido, é imperativo que os enfermeiros, como membros fundamentais das equipes da APS, estejam adequadamente preparados para atender às minorias sexuais e de gênero, garantindo um cuidado integral que respeite e atenda às suas realidades.

Compreender a diversidade como fator fundamental para a promoção da saúde em seu sentido mais amplo destaca a importância da equidade no SUS. No Brasil, a equidade é um princípio essencial para a promoção da justiça social, pois vai além da igualdade formal de acesso, buscando garantir que todas as pessoas tenham suas necessidades atendidas conforme suas especificidades. Isso implica tratar de forma diferenciada as populações em situação de maior vulnerabilidade, assegurando que políticas e ações de saúde contribuam para reduzir desigualdades e promover justiça social^(6)^. Nesse sentido, a APS, ao oferecer cuidado contínuo e coordenado, desempenha um papel fundamental na diminuição das iniquidades em saúde^(7)^.

As Políticas de Promoção da Equidade em Saúde no SUS consistem em programas e ações governamentais voltados para o respeito à diversidade e para o atendimento integral. O objetivo é eliminar disparidades injustas entre diferentes grupos populacionais, considerando variáveis como classe social, etnia, gênero e orientação sexual^(6)^. O Brasil tem buscado promover a diversidade cultural e os direitos sexuais e de gênero por meio de políticas, programas e diretrizes, como o Plano Nacional de Políticas para as Mulheres, Programas de Educação Inclusiva, legislação antidiscriminatória, apoio a eventos LGBTQIA+, programas de capacitação para profissionais de saúde e educação, fortalecimento das Políticas de Saúde Integral LGBTQIA+ e incentivo a ambientes de trabalho inclusivos.

Essas iniciativas refletem o compromisso do Estado com a diversidade e a inclusão. No entanto, sua efetividade ainda representa um desafio, uma vez que a implementação e o cumprimento dessas políticas variam em diferentes regiões do país. Para avançar, é essencial o engajamento contínuo do governo, da sociedade civil e dos indivíduos, a fim de promover uma cultura de respeito e igualdade.

Dessa forma, compreender as representações sociais que permeiam o cotidiano e as práticas de cuidado é crucial para promover a equidade. A interprofissionalidade constitui uma estratégia essencial para assegurar que a formação e a atuação dos profissionais de saúde estejam alinhadas ao princípio da equidade e aos objetivos da Agenda 2030.

## REFLEXÕES E DISCUSSÕES

A atenção integral à saúde da população LGBTQIA+ vai além do conhecimento técnico-científico. Ela exige uma compreensão crítica das dimensões sociais, culturais e políticas que influenciam tanto o acesso quanto a qualidade do cuidado.

No Brasil, a Política Nacional de Saúde Integral LGBT (PNSI-LGBT), instituída em 2011, tem como objetivo reduzir desigualdades e promover respeito e acolhimento^(7)^. No entanto, sua implementação ainda enfrenta desafios significativos devido ao impacto persistente de estigmas sociais e práticas hetero-cis-normativas nos serviços de saúde.

As desigualdades enfrentadas pela população LGBTQIA+ estão enraizadas em processos culturais e históricos que perpetuam desigualdades estruturais. Para que a formação em enfermagem contribua efetivamente para a promoção da equidade, é essencial que os currículos abordem de forma crítica as influências socioculturais e históricas sobre gênero e sexualidade, visto que normas sociais e representações culturais moldam não apenas as percepções de saúde e doença, mas também o próprio processo de formação e atuação dos profissionais de saúde^(8)^.

Uma formação em enfermagem baseada em princípios interprofissionais é fundamental para superar a fragmentação na educação em saúde^(9)^. Essa abordagem amplia a compreensão dos desafios enfrentados pelas minorias sexuais e de gênero, preparando profissionais aptos a oferecer um cuidado inclusivo e equitativo.

Estudos mostram que o processo formativo muitas vezes incorpora perspectivas hetero-cis-normativas, limitando a capacidade dos profissionais de oferecer um atendimento verdadeiramente equânime. Para romper com essas limitações, é essencial que os currículos de enfermagem questionem as influências culturais e sociais sobre gênero e sexualidade, enfatizando como as normas sociais impactam a saúde das minorias e capacitando os futuros profissionais a responder a essas demandas de forma sensível e inclusiva^(10-12)^.

### Cuidado Integral e Diversidade LGBTQIA+: Desafios e Estratégias

O cuidado integral à saúde da população LGBTQIA+ demanda uma abordagem abrangente que não se limite ao conhecimento técnico, mas que também incorpore uma compreensão crítica das dimensões sociais, culturais e políticas que impactam o processo saúde-doença, bem como o acesso e a qualidade do atendimento. Esse cuidado deve ser orientado pela ideia de integralidade, que, para além do foco na doença, reconhece e responde à complexidade das necessidades de cada pessoa, respeitando suas especificidades e particularidades em diferentes contextos de vida.

Para melhorar o entendimento e a acessibilidade, é fundamental disseminar conceitos mais amplos sobre diversidade de gênero e sexualidade, incluindo o termo “cisgênero” (ou “cis”), essencial para que os profissionais compreendam o impacto da lógica hetero-cis-normativa na prática clínica e no acolhimento de identidades não hegemônicas. A adoção de práticas como o uso de linguagem neutra e o respeito ao nome social revela-se crucial para garantir um atendimento que respeite a individualidade e a dignidade das pessoas LGBTQIA+^(8,13)^. No entanto, essas práticas muitas vezes são negligenciadas, demonstrando o quanto o cuidado integral depende de uma formação que transcenda normas sociais limitantes.

Estudos nacionais e internacionais, como os realizados no Brasil e na Austrália, indicam que a população LGBTQIA+ enfrenta barreiras significativas no acesso aos serviços de saúde, incluindo experiências de discriminação e distanciamento por parte dos profissionais, reflexo de preconceitos e atitudes desrespeitosas^(14,15)^.

Tais dificuldades estão enraizadas em um histórico de desigualdades estruturais e práticas sociais excludentes, o que demanda dos profissionais de saúde um entendimento aprofundado de como as normas culturais e sociais moldam percepções de saúde e práticas assistenciais. Por exemplo, um estudo em Santa Catarina, que investigou as experiências de pessoas trans e equipes de enfermagem em diferentes níveis de atenção, revelou que a formação deficitária em diversidade sexual e de gênero contribui para práticas de cuidado que reproduzem exclusões^(11)^.

O currículo acadêmico, muitas vezes moldado por uma visão hetero-cis-normativa, limita a compreensão das diversas formas de ser e estar no mundo, impactando diretamente a capacidade de oferecer um cuidado que seja realmente integral e equânime. Para enfrentar as iniquidades em saúde, é imprescindível que a formação de enfermeiros e outros profissionais de saúde incorpore um entendimento crítico das influências culturais, religiosas e históricas que afetam a saúde de minorias sexuais e de gênero. A inclusão de conteúdos sobre a trajetória histórica de opressões e sua relação com a saúde é uma estratégia educativa poderosa para promover práticas que busquem equidade e humanização^(13,16)^.

Esse enfoque crítico é essencial, pois estudos mostram que a formação tradicional, impregnada de normas hetero-cis-normativas, ainda falha em preparar adequadamente os profissionais para o atendimento inclusivo e equânime de minorias. Ademais, uma formação que contemple a diversidade sexual e de gênero e inclua experiências práticas com grupos historicamente marginalizados é essencial para que o cuidado integral se concretize.

A integração de práticas de contato intergrupal, especialmente com minorias sexuais e de gênero, é uma estratégia para promover empatia e compreensão das realidades distintas que configuram as experiências desses grupos. A perspectiva interseccional, que considera a sobreposição de opressões, como racismo, sexismo e LGBTfobia, também é indispensável para que os profissionais desenvolvam uma visão integral do cuidado, reconhecendo e respeitando as múltiplas dimensões identitárias e de vida das pessoas.

Assim sendo, o cuidado integral exige que os profissionais de saúde estejam preparados para entender e responder às necessidades de cada indivíduo de forma contextualizada e crítica. Isso demanda uma formação que abra espaço para a construção de práticas de acolhimento e cuidado baseadas em empatia, respeito e inclusão, capazes de superar as limitações impostas pelas normas sociais tradicionais e promover uma assistência em saúde que seja realmente inclusiva e equitativa.

### Educação interprofissional: perspectivas inclusivas no cuidado à população LGBTQIA+

A interprofissionalidade diz respeito ao desenvolvimento de uma prática colaborativa e integrada entre diferentes profissões de saúde. Assim, por meio da educação interprofissional, as competências essenciais para a atuação em saúde vão além das habilidades técnicas, abrangendo também a capacidade de ouvir, dialogar e compartilhar responsabilidades^(9)^.

Essa abordagem prepara os profissionais para entenderem que o cuidado integral, especialmente para populações vulneráveis, como a LGBTQIA+, demanda o reconhecimento e a valorização dos saberes e experiências. Além disso, promove a interdependência e a complementaridade entre os profissionais, evitando a subordinação e impedindo que uma única área domine as abordagens de cuidado.

A interdisciplinaridade, por sua vez, destaca a importância de integrar conhecimentos que transcendam as fronteiras de cada disciplina, sendo fundamental para enfrentar problemas de saúde complexos inseridos em contextos culturais, sociais e econômicos amplos^(9)^. A saúde das populações LGBTQIA+, por exemplo, exige uma compreensão crítica das questões de gênero, sexualidade e equidade. Para promover práticas de saúde verdadeiramente equitativas e inclusivas, a formação precisa estimular uma análise crítica das normas sociais e culturais que sustentam preconceitos e desigualdades, muitas vezes enraizados em paradigmas hetero-cis-normativos^(11,13)^.

Dessa maneira, a interprofissionalidade e a interdisciplinaridade, em conjunto, se alinham às Diretrizes Curriculares Nacionais (DCNs) e à Política Nacional de Educação Permanente em Saúde (PNEPS), uma vez que essas políticas incentivam as universidades a criarem espaços para o desenvolvimento de uma prática educativa reflexiva e dialógica, que permita o enfrentamento das lacunas de formação por meio do diálogo entre diferentes saberes e práticas.

A implementação de programas como o VER-SUS (2004), Pró-Saúde (2007) e PET-Saúde (2010) exemplifica o papel da interprofissionalidade em desafiar visões estereotipadas e preconceituosas, promovendo uma formação focada na equidade. Esses programas incentivam a interação entre diferentes áreas do conhecimento e práticas de saúde, preparando estudantes e profissionais para atender às necessidades da população LGBTQIA+ com empatia, competência técnica e sensibilidade relacional. Ao integrar equipes diversas, fortalecem a capacidade dos futuros profissionais de oferecer um cuidado mais inclusivo e integral^(13)^.

Além disso, é fundamental refletir sobre o papel da cultura na perpetuação de processos excludentes e segregadores, oriundos majoritariamente do cristianismo, que moldou normas culturais que, muitas vezes, sustentam formas de opressão e afetam as percepções sociais sobre sexualidade e gênero. Assim, nota-se que superar as iniquidades em saúde exige também a desconstrução dessas normas culturais, incorporando uma compreensão crítica das influências sociais e culturais na saúde das minorias^(13)^.

Para cumprir as metas dos ODS da Agenda 2030, é essencial que a formação de profissionais de saúde incorpore, desde a graduação, as dimensões interprofissionais. Esse fortalecimento é especialmente relevante na APS, onde os profissionais frequentemente têm o primeiro contato com populações vulneráveis e marginalizadas, que requerem um cuidado contínuo e abrangente.

A prática educativa interprofissional deve ser emancipada e orientada para a justiça social, possibilitando que futuros profissionais compreendam o impacto das condições sociais e culturais na saúde das pessoas e saibam responder de forma inclusiva. As universidades, nesse sentido, devem ser espaços de construção de práticas transformadoras, com currículos que formem profissionais comprometidos com a inclusão e a equidade, criando uma base sólida para um sistema de saúde que atenda a todos.

Essa abordagem está diretamente alinhada aos ODS, que destacam a importância de uma formação capaz de responder às necessidades globais de saúde. Para oferecer um cuidado que considere a integralidade do ser humano, as universidades precisam promover ambientes de diálogo e práticas interprofissionais, estimulando o desenvolvimento de uma formação crítica e voltada à justiça social. Esse modelo de formação transforma a prática profissional e contribui para um sistema de saúde mais inclusivo e equitativo, atendendo, em especial, às necessidades da população LGBTQIA+.

A diversidade cultural, que é central para a promoção da saúde, deve ser abordada nas políticas públicas brasileiras, garantindo o acesso equitativo à saúde para todas as populações, com especial atenção às minorias de gênero e sexuais. A enfermagem, nesse contexto, exerce um papel essencial, pois a formação superior na área tem evoluído para responder a cada conjuntura histórica, estabelecendo relações terapêuticas e interprofissionais de cuidado que exigem uma construção crítica e criativa do conhecimento. Esse processo deve incluir um diálogo constante entre o indivíduo, a família e os demais profissionais da equipe de saúde.

É igualmente importante reconhecer que o indivíduo é parte de uma sociedade, o que torna imprescindível integrar a saúde coletiva à formação em enfermagem. Compreender que os fenômenos sociais se desenham nas interações e no papel que desempenhamos no mundo aumenta a necessidade de as universidades discutirem amplamente sua função social. Essa reflexão permite o desenvolvimento de alternativas educacionais emancipadoras. Currículos que ignoram a complexidade e a diversidade social comprometem a compreensão ampliada do processo saúde-doença e limitam a capacidade de trabalho em equipe, reduzindo a resolutividade dos cuidados^(13,16)^.

Portanto, os conteúdos curriculares precisam ser abordados de forma crítica e contextualizada, promovendo uma formação que responda às demandas sociais e assegure o direito à saúde para todos, especialmente para as populações mais vulneráveis. Essa necessidade reforça a importância da interprofissionalidade e da interdisciplinaridade na formação em enfermagem, preparando profissionais capazes de atuar de maneira integrada e colaborativa. Com essa abordagem, torna-se possível promover uma saúde global e equitativa, em consonância com os ODS da Agenda 2030.

## CONSIDERAÇÕES FINAIS

A formação interprofissional e interdisciplinar em enfermagem é essencial para transcender os modelos fragmentados de educação em saúde, promovendo um cuidado integral e inclusivo que responda às demandas das minorias sexuais e de gênero. Ao integrar saberes e práticas de diversas áreas, essa abordagem amplia a capacidade dos profissionais para enfrentar as complexidades sociais, culturais e éticas envolvidas no cuidado da população LGBTQIA+. Dessa forma, os futuros profissionais de enfermagem são preparados para oferecer um atendimento que respeite a diversidade e assegure a equidade na saúde.

Incorporar essas dimensões no currículo de enfermagem é essencial para garantir uma formação sensível às necessidades específicas dessa população, alinhando-se aos ODS da Agenda 2030, em especial nas metas relacionadas à educação de qualidade, igualdade de gênero e redução das desigualdades. Isso fortalece a prática profissional e contribui para a construção de um sistema de saúde mais justo e equitativo, capaz de oferecer um cuidado respeitoso e humanizado.

Portanto, é necessário que os currículos de enfermagem incluam práticas educativas interprofissionais que abordem as necessidades de saúde dessa população. A formação deve incentivar a colaboração entre diferentes áreas do conhecimento e considerar as múltiplas realidades das minorias sexuais e de gênero, para que se alcance a equidade em saúde. Dessa forma, o cuidado integral será efetivamente concretizado no contexto do SUS, respeitando as especificidades e promovendo a saúde de forma inclusiva.
